# On-Table Hypoxic Arrest: A Comparative Examination of Peripartum Cardiomyopathy and Pre-eclampsia

**DOI:** 10.7759/cureus.83605

**Published:** 2025-05-06

**Authors:** Clara Tong, Aaron Kong, Farida Ithnin, Ban Leong Sng

**Affiliations:** 1 Department of Anaesthesiology and Surgical Intensive Care, Changi General Hospital, Singapore, SGP; 2 Department of Women's Anaesthesia, KK Women's and Children's Hospital, Singapore, SGP

**Keywords:** cardiac arrest, cardiomyopathy, hypoxia, peripartum, pre-eclampsia

## Abstract

Pre-eclampsia (PE) and peripartum cardiomyopathy (PPCM) are two types of complications that can occur in pregnancy and the peripartum period. We present the successful management of a pre-eclamptic patient with severe, undifferentiated pulmonary oedema in pregnancy.

Our patient presented at 35 + 5 weeks of gestational age with hypertension, delirium and severe hypoxaemia. She developed pulseless electrical activity after induction of general anaesthesia for a category one caesarean section. Resuscitation commenced, and a peri-mortem caesarean section was performed. Spontaneous circulation returned nine minutes after arrest. Post-operative evaluation in the intensive care unit revealed elevated natriuretic peptide levels, cardiomegaly and left ventricular ejection fraction of 20%-25%.

Pulmonary oedema associated with PE and PPCM may have similar clinical features but may be distinguished with a targeted bedside echocardiogram. This guides disease-specific management and prognostication. Strategies to prevent severe hypoxaemia during laryngoscopy and intubation may also be considered. These cases require expert care and good inter-disciplinary team dynamics to ensure a favourable patient outcome.

## Introduction

Peripartum cardiomyopathy (PPCM) is classically defined as an idiopathic cardiomyopathy, with a left ventricular ejection fraction (LVEF) of <45%, occurring towards the end of pregnancy or in the months following delivery, abortion or miscarriage [1]. Pre-eclampsia (PE) refers to the new onset of hypertension and proteinuria or the new onset of hypertension plus significant end-organ dysfunction with or without proteinuria in a previously normotensive patient, typically after 20 weeks of gestation or postpartum. While worldwide incidence of PPCM was found to be between 0.006% and 0.98% [2], emerging evidence has identified an increased prevalence of hypertensive disorders in women with PPCM, reporting the incidence of PE in this group of patients to be approximately 22% [3], which was more than quadruple the 5% average worldwide background rate of PE in pregnancy. PE is also a risk factor for PPCM, which has common underlying pathophysiological pathways [4,5]. In patients with severe PE and symptoms of heart failure, PPCM should always be listed as a differential diagnosis, because differences in management between PE with vs. PE without PPCM will have an impact on the patient's outcome. With the patient’s consent, we present the successful management of a case of severe, undifferentiated pulmonary oedema in pregnancy, against the background of known severe PE.

## Case presentation

Our patient was a 35-year-old, obese (body mass index 34 kg/m^2^), multiparous lady. She had a history of myomectomy done in 2017 and one previous caesarean delivery. Her current pregnancy antenatal history was significant for gestational diabetes on diet control, as well as proteinuria and hypertension in keeping with her PE diagnosis that was made at 35 weeks of gestation, which was treated with oral labetalol and methyldopa. She was also on nifedipine, utrogestan and weekly progesterone injections. She presented at 35 + 5 weeks of gestational age with acute respiratory distress and was emergently transferred to our centre for further management. Other antenatal records were not accessible at the point of presentation, as her antenatal follow-up and investigations were all done at another private hospital under a different electronic medical record system. The only information we had when she was transferred over to our institution was that her infective screen was non-reactive, blood group was A+ with a haemoglobin level of 13.4 g/dL at booking, proteinuria 4+ was detected at 35 + 1 weeks with elevated liver enzymes (aspartate transaminase 45 and alanine transaminase 78) and intramuscular dexamethasone had been given.

Initial assessment revealed an obtunded patient with a borderline Glasgow Coma Scale (GCS) of E2V2M5. The patient was severely tachypnoeic (respiratory rate 40/minute) and hypoxaemic (peripheral oxygen saturation, SpO_2_, 58% on non-rebreather mask). Diffuse inspiratory crepitations were heard on auscultation. Sinus tachycardia (heart rate 137/minute), with hypertension (blood pressure, BP, 149/78 mmHg), was also noted. Arterial blood gas (ABG) analysis showed severe respiratory failure (Table 1). A 4-g loading dose of magnesium sulphate was immediately commenced and followed by an infusion of 1 g/hour. Continuous foetal cardiotocography was commenced on admission to the labour ward. At that time, there were no concerns regarding foetal well-being in terms of heart rate monitoring. The presence of foetal heartbeat was confirmed by bedside ultrasound examination. After a quick multi-disciplinary discussion, the patient was listed for category one caesarean section. Just before induction of anaesthesia, a Doppler was done to ensure the presence of foetal heart rate as a confirmation to proceed with caesarean surgery.

**Table 1:** Arterial blood gas analysis at time of presentation pCO_2_: partial pressure of carbon dioxide; pO_2_: partial pressure of oxygen; HCO_3_: bicarbonate; sO_2_: sulphur dioxide; K: potassium; Na: sodium; iCa: ionised calcium; Hct: haematocrit; Hb: haemoglobin

Parameter	Value	Normal range
pH	7.299	7.35-7.45
pCO_2_	53.2 mmHg	35-45 mmHg
pO_2_	43 mmHg	80-100 mmHg
HCO_3_	22.2 mmol/L	22-26 mEq/L
Base excess	-5 mmol/L	-2 to +2
sO_2_	68%	≥95%
Na	137 mmol/L	135-150 mmol/L
K	5.5 mmol/L	3.5-5 mmol//L
iCa	1.22 mmol/L	1.12-1.40 mmol/L
Hct	46%	36%-50%
Hb	15.6 g/dL	11.6-16.6 g/dL

The impaired gas exchange as a result of severe pulmonary oedema impaired pre-oxygenation efforts, with a maximum attainable SpO_2_ of about 80% despite 100% oxygen via a tight-fitting face mask at flows of 15 L/minute. Rapid sequence induction was performed with thiopentone 375 mg and succinylcholine 100 mg. The patient was intubated with a size 7.5 standard endotracheal tube (ETT) using a McGrath videolaryngoscope (Medtronic, Minneapolis, MN), which showed a Cormack-Lehane grade one view of the larynx. Copious frothy secretions were seen in the ETT and oropharynx after intubation; hence, suctioning was performed immediately. During this time, SpO_2_ dropped further to 50%, and there was worsening bradycardia, which eventually led to pulseless electrical activity (PEA) arrest. The patient was resuscitated according to Advanced Cardiac Life Support protocol. The decision was made for peri-mortem caesarean section to improve maternal resuscitation efforts, and this commenced two minutes after arrest, with Pfannenstiel skin incision and lower transverse uterine incision.

During resuscitation efforts, the foetus had turned to a transverse back-down position, which resulted in the obstetric team delivering the baby breech with some difficulty. A 2.57-kg baby girl was delivered, and the umbilical cord gas results are shown in Table 2. Neonatal resuscitation was required as the infant was pink but limp with no cry on delivery, noted to be gasping and bradycardic at 100 beats/minute on auscultation; hence, she was started on intermittent positive pressure ventilation 20/5 with 100% oxygen immediately. APGAR was six at one minute, and the infant was subsequently intubated at two minutes of life as she was still gasping. At five minutes of life, APGAR was nine, the infant's heart rate was 181 beats/minute, SpO_2_ was 92% and the infant was pink and moving spontaneously. She was transferred to the neonatal intensive care unit (ICU) at eight minutes of life, and oxygen supplementation was weaned down to 40%.

**Table 2:** Umbilical cord gas results

Parameter	Arterial	Venous
pH	6.766	6.932
Lactate	11.6	8.5
Base excess	Not reflected	Not reflected

Ventilation for the patient was continued at 100% oxygen to address hypoxia as the primary cause of the PEA arrest. Other causes that were suspected included hyperkalemia, and one cycle of hyperkalemia treatment (10% calcium gluconate 10 mL, insulin 10 IU and dextrose 50% 20 mL) was given. The patient regained spontaneous circulation three minutes after delivery (eight minutes after arrest), with three cycles of cardiopulmonary resuscitation and two 1-mg boluses of adrenaline administered intravenously, four minutes apart. Arterial and central venous catheters were subsequently sited. ABG analysis was performed to exclude significant acidosis and electrolyte abnormalities, and the results are shown in Table 3. Although the patient did not require inotropic support, oxygenation was borderline, with SpO_2_ 88%-90% and a partial pressure of oxygen 89 mmHg despite ventilation with 100% oxygen at high flows of 15 L/minute. Furosemide was given to aid diuresis. The caesarean section concluded uneventfully, with blood loss of approximately 600 mL. She was transferred to the ICU for post-operative monitoring. The patient’s vital signs at various stages during the event are shown in Table 4.

**Table 3:** ABG analysis 21 minutes after return of spontaneous circulation pCO_2_: partial pressure of carbon dioxide; pO_2_: partial pressure of oxygen; HCO_3_: bicarbonate; sO_2_: sulphur dioxide; K: potassium; Na: sodium; iCa: ionised calcium; Hct: haematocrit; Hb: haemoglobin; ABG: arterial blood gas

Parameter	Value	Normal range
pH	7.07	7.35-7.45
pCO_2_	72.7 mmHg	35-45 mmHg
pO_2_	89 mmHg	80-100 mmHg
HCO_3_	21.1 mmol/L	22-26 mEq/L
Base excess	-9 mmol/L	-2 to +2
sO_2_	92%	≥95%
Na	135 mmol/L	135-150 mmol/L
K	4.4 mmol/L	3.5-5 mmol//L
iCa	1.21 mmol/L	1.12-1.40 mmol/L
Hct	38%	36%-50%
Hb	12.6 g/dL	11.6-16.6 g/dL

**Table 4:** Vital signs at various stages during the event ROSC: return of spontaneous circulation; ICU: intensive care unit; SpO_2_: peripheral oxygen saturation; FiO_2_: fraction of inspired oxygen

Event	Blood pressure (mmHg)	Heart rate (bpm)	SpO_2_ (%)
At induction	145/95	118	80+ (after preoxygenation)
Immediately after induction	90/60	130	80+ (before intubation)
Three minutes after induction	Unrecordable	30+	50
Immediately after ROSC	150/96	120	80 (FiO_2_ 100%)
Before transfer to ICU	120/65	122	87 (FiO_2_ 100%)

**Figure 1:**
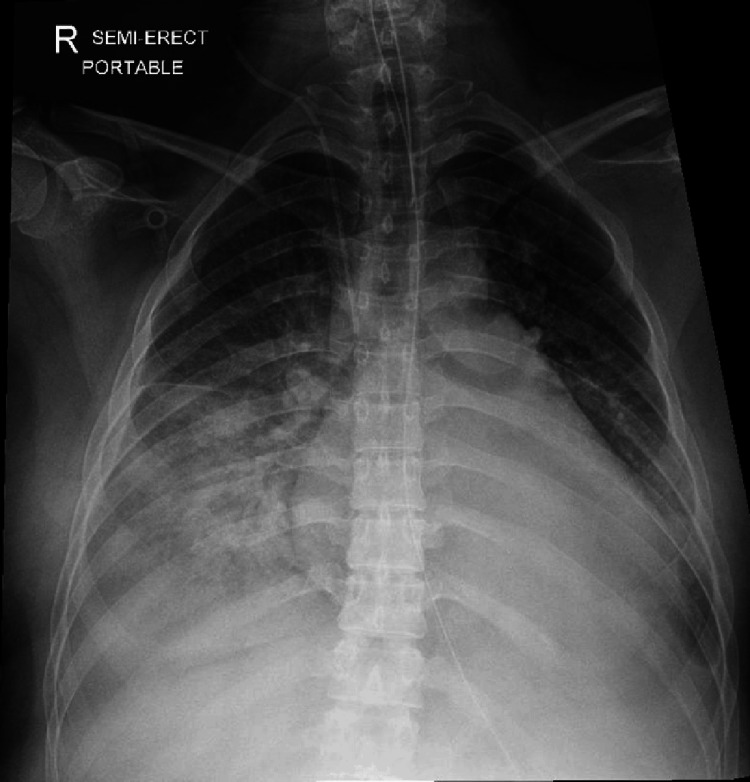
Chest radiograph taken shortly after admission to ICU Although cardiomegaly is conventionally diagnosed on a posterior-anterior chest radiograph, this anterior-posterior film suggests cardiomegaly even after considering projection differences, especially in this context of features indicative of pulmonary oedema (severe air space shadowing in bilateral lower zones and upper lobe diversion) ICU: intensive care unit

**Figure 2:**
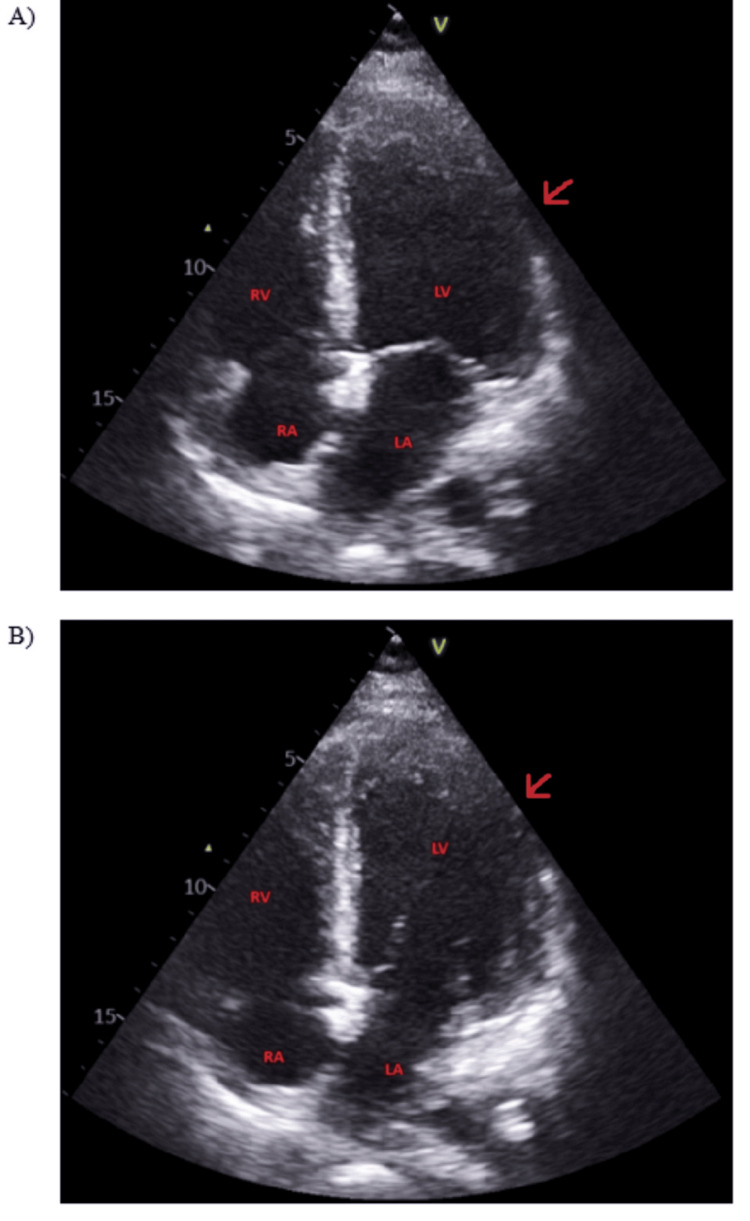
Transthoracic echocardiography performed shortly after ICU admission. The apical four-chamber view in (A) systole and (B) diastole The arrow in (A) shows severe global LV hypokinesia with no LV hypertrophy. The arrow in (B) comparing the LV size in diastole shows a severely impaired LV estimated ejection fraction of 20%-25% (visually estimated on a formal transthoracic echocardiography). In the same study, LV internal diameter in end-diastole was 52 mm, LV internal diameter in end-systole 44 mm, and LV fractional shortening was 15% ICU: intensive care unit; LV: left ventricular; RV: right ventricular; LA: left auricular; RA: right auricular

A cardiology referral was made in the ICU. Natriuretic peptide levels were elevated. Serial cardiac troponins were raised initially and measured until a falling trend was demonstrated. Chest radiograph showed an enlarged heart with severe air space shadowing in bilateral lower zones (Figure 1). Trans-thoracic echocardiography revealed severely impaired left ventricular (LV) ejection fraction of 20%-25%, on a background of severe global LV hypokinesia with no LV hypertrophy or pulmonary hypertension (Figure 2). There was no evidence of right ventricular dysfunction or strain. Loop diuretics were continued, and ventilator support was gradually weaned. The patient transiently required haemodynamic support with dobutamine infusion. No features of haemolysis, elevated liver enzymes or low platelet count syndrome were detected. She demonstrated good neurological recovery, with GCS of E4VTM6 and appropriate response to questions by nodding, gestures and writing by 12 hours after the event. She was extubated the next morning, after which she had full neurological recovery. She was then transferred to a tertiary cardiac centre for further management. Carvedilol, enalapril, spironolactone and ivabradine were started, and diuresis was continued. She was subsequently discharged home five days later.

## Discussion

This case highlights the challenges of a patient with known severe PE who presented in extremis. There was limited time for assessment and optimisation before caesarean delivery. Although there were no signs of foetal distress on the cardiotocography done in the labour ward, it was felt that an expedited delivery would aid maternal resuscitation. Acute pulmonary oedema is a leading cause of death in women with PE, occurring in up to 3% of cases [6]. This is a result of reduced plasma colloid oncotic pressure, altered endothelial permeability and possible LV hypertrophy with diastolic dysfunction associated with PE [6,7]. These patients are also more likely to have a family history of hypertension, history of hypertensive disorders in previous pregnancies and tachycardia at presentation. In contrast, the presence of cardiomegaly with reduced LVEF, left atrial hypertrophy, QRS abnormalities, T wave inversion and atrial fibrillation, especially in the setting of smoking and twin pregnancy, is suggestive of PPCM. This distinction is important because it impacts subsequent management and prognostication: mortality among patients with PPCM was 17%, compared with 0% for those with hypertensive heart failure of pregnancy [8].

Therefore, in this situation where both pathologies can co-exist, it is important to rapidly assess underlying cardiac function before attributing the physiological derangements to PE alone. A valuable tool to do so would be a targeted bedside echocardiogram, in the form of a point-of-care ultrasound (POCUS) examination. However, POCUS requires the operator to be trained and may be challenging to get good images, considering the body habitus of the pregnant patient, when done in an emergency, non-optimised environment. Other cardiac output monitoring can also be used, such as a non-invasive cardiac output monitor using bioimpedance technology or arterial waveform analysis. These modalities can help identify complications and guide fluid management, and therapy can be tailored to address both entities.

In severe PE, the treatment would be for delivery of the foetus, in addition to magnesium sulphate infusion, fluid restriction and BP control. However, if PPCM were concomitant, the choice of anaesthetic agents may then be optimised, and early inotropic support may be instituted in anticipation of myocardial depression associated with the induction of general anaesthesia. In hindsight, arterial line insertion before induction of general anaesthesia might have been beneficial for beat-to-beat monitoring of BP, which might have allowed for earlier detection of collapse after induction. However, it would not have been prudent to delay induction for invasive line insertion if the first attempt was met with difficulty. Early involvement of the cardiologist and the institution of specific therapy, such as bromocriptine, oral heart failure drugs, anticoagulation, vasorelaxing agents and a diuretic regimen [1], can also be planned.

In view of the patient’s clinical presentation, difficulty with pre-oxygenation and rapidity of deterioration during intubation efforts, hypoxia was the most likely cause for the eventual cardiac arrest. It is therefore worthwhile exploring strategies to prevent severe hypoxaemia during intubation attempts. In a patient with cardiogenic pulmonary oedema, preload and afterload reduction using a combination of morphine, diuretics and nitrates reduces extravascular lung water and may improve gas exchange at the alveoli [9]. The use of non-invasive ventilation is also associated with rapid and significant improvement in respiratory and haemodynamic parameters through alterations in respiratory mechanics as well as preload and afterload reduction [10,11]. However, NIV in pregnancy also poses an increased risk of aspiration and should only be used for alert and conscious patients with good respiratory drive, stable haemodynamics and no severe acid-base disturbances. Hence, this patient would not have been a good candidate.

Although the use of high-flow nasal cannula (HFNC) for apnoeic oxygenation has been shown to be safe and effective in prolonging safe apnoea time in obstetric patients [12,13], evidence for its use in the setting of respiratory failure is less clear. A recent meta-analysis showed that the use of HFNC for apnoeic oxygenation was at least non-inferior to standard of care in the prevention of severe hypoxaemia during tracheal intubation, with particular benefit in the subgroup of patients with mild hypoxaemia (partial pressure of oxygen/fraction of inspired oxygen ratio >200 mmHg) [14]. However, this was limited by significant clinical heterogeneity in study methodology as well as outcome measures. In a patient with significant intrapulmonary shunting secondary to pulmonary oedema, alveolar recruitment is essential. The above strategies may be useful in achieving this and are worthy of consideration, especially given the lack of time and alternatives in this patient.

Effective teamwork and coordination between multidisciplinary teams are vital in managing this unstable parturient. The acuity and severity of presentation afforded little time for discussion and management planning. To improve team dynamics and delivery of emergency care, the use of simulation would be useful to increase awareness and competency in maternal resuscitation among team members. The experience gained from simulation training will be applicable beyond this single case, as different simulation scenarios can be played out to achieve the required learning objectives. Such programs have been implemented in several centres, with very good participant feedback [15,16].

## Conclusions

In conclusion, pregnant patients may present with PE with or without PPCM. The distinction between the two is important to make, as it ultimately impacts management and prognostication. Regardless, the complex of PE and PPCM presents a unique challenge to the anaesthetist, requiring expert care and good inter-disciplinary team dynamics to ensure a favourable patient outcome. This case highlights the role of POCUS in distinguishing PPCM from hypertensive heart failure of pregnancy, guiding anaesthetic and medical decisions during critical maternal events.
